# Treatment-related problems in neonates receiving parenteral nutrition: risk factors and implications for practice

**DOI:** 10.1186/s12887-023-04477-1

**Published:** 2024-01-03

**Authors:** Amal Akour, Lobna Gharaibeh, Omar El Khatib, Khawla Abu Hammour, Noor AlTaher, Salah AbuRuz, Muna Barakat

**Affiliations:** 1 Department of Pharmacology and Therapeutics, College of Medicine and Health Sciences, United Arab Emirates University, Al Ain, UAE; 2 Department of Biopharmaceutics and Clinical Pharmacy, School of Pharmacy, The University of Jordan, Amman, Jordan; 3 Biopharmaceutics and Clinical Pharmacy Department, Faculty of Pharmacy, Al-Ahliyya Amman University, Amman, Jordan; 4 Department of Clinical Pharmacy and Therapeutics, School of Pharmacy, Applied Science Private University, Amman, Jordan

**Keywords:** Parenteral nutrition, Treatment-related problems, Neonates, Intensive care unit

## Abstract

**Objectives:**

Parenteral nutrition (PN) can be associated with several treatment-related problems (TRPs) and complications in neonatal settings. Thus, understanding the extent and type of these problems and related factors is pivotal to prevent negative consequences of these preparations. Thus, the aim of this study is to assess factors affecting TRPs in neonatal patients receiving PN.

**Methods:**

This was a retrospective chart review of neonates receiving PN in NICU and other wards. We collected their demographics, and laboratory workup. TRPs related to PN preparations as well as their pharmacotherapy were the primary outcomes.

**Results:**

Medical charts of 96 neonate were reviewed. The most encountered TRPs related to patients’ pharmacotherapy were the lack of frequent monitoring (34.2%) and low dose (17.5%). For PN-related TPRs, a mismatch between patients’ nutritional needs and PN composition was observed in third of the patients. Statistically significant positive correlations between number of medications during hospital stay and number of reported TRPs [(*r* = 0.275, *p* < 0.01) and (*r* = 0.532, *p* < 0.001)] were observed.

**Conclusion:**

In neonates who receive parenteral nutrition (PN), TRPs are often observed. These problems primarily arise from issues in patients’ pharmacotherapy, namely monitoring and dosing. Identifying the risk factors for these TRPs emphasizes the full and effective integration of clinical pharmacists into the healthcare team, which can serve as a potential preventive strategy to lower the occurrence of TRPs.

## What is known?

- Parenteral nutrition (PN) in neonates is associated with several treatment-related problems (TRPs) such as metabolic complications, infections, incompatibility issues and/or nutritional imbalances.
- Vigilant monitoring, strict aseptic techniques and involving pharmacists in the care team is crucial to manage and/or prevent these TRPs.

## What is new?

- Inadequate monitoring, suboptimal doses, and nutritional imbalances are very common TRPs in this population.
- The frequency of TRPs is proportionally associated with the duration of hospitalization, number of medications, as well as renal and liver dysfunction.
- Individualized therapy based on neonatal nutritional needs, underlying conditions, and response to therapy, is crucial and yet a novel investigational area.

## Introduction

Parenteral nutrition (PN) is a crucial therapeutic approach utilized for a range of conditions in adults, and pediatrics [1]. These involve ailments of the gastrointestinal tract (GIT), such as intestinal obstruction, inflammatory bowel disease (IBD), as well as severe malnutrition and anticipated long-term starvation [1]. In neonatal settings, PN plays a vital role in providing essential nutrients to infants who are unable to tolerate enteral feeding [2, 3]. However, it also presents unique challenges related to drug administration due to complexities based on many factors, such as gestational age and body weight [2]. The proper application of this complicated therapy attempts to maximize therapeutic benefit while reducing the likelihood of unfavorable outcomes. PN formulations must be sterile and contain stable and compatible components due to the complexity of their composition and direct delivery into the bloodstream, which is necessary to ensure the safety of patients receiving PN therapy [1, 4, 5]. Patients may suffer injury from an intravenous infusion that is incompatible, unstable, or contaminated, including major morbidity and even mortality [6, 7]. As a result, PN formulations must be prepared using strict aseptic processes in accordance with established guidelines for pharmaceutical compounding [7, 8]. Even though PN is recognized as a high-alert medicine, a limited number of organizations have protocols to prevent dispensing errors and PN-related patient adverse events [8]. In fact, both the PN preparations as such and the therapy can potentially cause complications and treatment-related problems (TRPs).

Treatment-related problems, especially in neonatal patients, receiving PN represent a significant concern in healthcare settings [1]. The administration of medications to this vulnerable population requires utmost precision and attention to ensure optimal patient outcomes [1]. Understanding the factors that contribute to the occurrence of TRPs is crucial for the development and implementation of effective strategies for prevention and mitigation [9].

Furthermore, the frequency and type of TRPs within this context will provide valuable insights into the extent of the problem within neonatal populations [2]. Identifying potential factors associated with these complications will enable healthcare professionals to tailor interventions specifically targeting these areas for improvement [10]. These factors can be related to problems in drug dosing, drug-drug interactions, or inappropriate administration. On the other hand, the problems may be related to medication choice, unnecessary drug therapy, side effects, or untreated diseases [10, 11].

Pharmacists are among the healthcare providers who could contribute significantly to this domain. They possess medical expertise which allows them to manage medication administration and preparation [7]. As part of PN team, they can prevent PN-related problems by providing nutrition support services and the provision of standard operating procedure upon administering and dispensing of PN procedures in neonatal settings [12, 13]. Pharmacists’ intervention enhanced nutritional support, weight attainment by low birthweight infants and reduced cost of care. This requires interdesciplinary collaboration with other healthcare professionals including nutritionists, nurses, and physicians, who are involved in this process [14]. Indeed, all the pharmacists' role is embedded under the umbrella of pharmaceutical care, which arranges their responsibilities to achieve the best health outcome for the patient [15]. Moreover, the pharmacy profession in PN settings, have been scaled up from conventional compounding and dispensing to a highly sophisticated handling, which is incorporate artificial intelligence and modern technology [7, 16, 17].

Therefore, addressing TRPs and understanding the associated factors may guide healthcare providers in adjusting medication management protocols and promoting safer practices when administering drugs via parenteral routes. Reducing or eliminating TRPs can improve overall patient safety, enhance treatment outcomes, and optimize resource utilization within neonatology units. This study investigates the frequency and potential factors correlated with TRPs in neonatal patients receiving PN. In addition, by identifying these factors, healthcare professionals can gain insights into the underlying causes of TRPs and devise interventions that address them comprehensively.

## Methods

### Settings and participants

This was a retrospective chart review, that included neonates who are on parenteral nutrition for any duration at the University of Jordan Hospital over four-month period. The study was approved by the Jordan University Hospital International Review Board (IRB) (IRB number: 109/2023). All patients who attended any hospital ward and received any type of PN were included in this study. Patients were excluded if they only received enteral nutrition.

The following data were collected: demographics such as age, gender, weight and height, and race. Laboratory workup was also obtained from patient’s medical files. PN indication and duration were also documented. PN was administered through central line in most of the cases (TPN), while peripheral route was used when there is partial PN that is used for short-time nutritional support (< 5 days). The osmolarity and pH were checked by the clinical pharmacist. The standard formula for the central PN contained 200 ml of 25% dextrose, 30 ml of 3% hypertonic saline, 150 ml of 10% Aminplasmal® (B. Braun Melsungen AG, Germany), 7.5 ml of 10 mEq KCl, and then distilled water was added to have a final volume of 500 ml. Intralipid® (Fresenius Kabi AB, Sweden) was started at a dose of 1g/kg daily. These percentages were then adjusted on individual bases.

TRPs were analyzed according to pre-defined classification adapted from Cipolle et al. [16] and Aburuz et al. [17].

### Statistical analysis

Data analysis was performed using the Statistical Package of Social Sciences (SPSS) version 24 (IBM, USA). Continuous variables were presented as mean ± standard deviation (SD) or median (Interquartile range), as appropriate, while categorical variables were described as frequency (percentages). The Spearman correlation coefficient was used to measure associations between TRPs and other variables. Then, those variables that were found significant as single predictors were included in multiple linear regression. Variables were checked for their independence, where tolerance values > 0.1 and Variance Inflation Factor (VIF) values were < 10 to indicate the absence of multicollinearity between the independent variables in regression analysis. None of the included variables showed multicollinearity, thus, none was eliminated. A *p*-value of < 0.05 was considered statistically significant.

## Results

### Demographic characteristics and laboratory workup.

A total of 96 subjects were screened for inclusion, all of which satisfied the inclusion criteria. Table 1 represents patients’ demographic characteristics. Among all study subjects, 44.8% (*n* = 43) were females while 55.2% (*n* = 53) were males with mean weight (± SD) of 1.56 ± 0.60 kg. All the study subjects were Jordanians, with the vast majority receiving their PN therapy within the first week of life (*n* = 94, 97.9%). More than two thirds of the neonates were admitted into NICU (77%, *n* = 74), while the remainder were hospitalized in different wards (23%, *n* = 22), with median duration of hospital stay (IQR) of 33 days (21.3–45.8). The majority were preterm (82.3%, *n* = 97) with half of them diagnosed with respiratory distress (RDS) (53.1%, *n* = 51) and sepsis occurred in almost half of infants (all of them were preterm neonates).

**Table 1:** Demographics and laboratory workup of neonates hospitalized upon birth (*n* = 96)

Variable	
Time of PN administration, n (%)	
Within first week of birth	94 (97.9)
After one week of birth	2 (2.1)
Weight (Kg), mean ± SD	1.56 ± 0.60
Height (cm); mean ± SD	39.5 ± 5.07
Gestational age (weeks); mean ± SD	31.8 ± 3.50
Gender, n (%)	
Males	43 (44.8)
Females	53 (55.2)
Ethnic Origin, n (%)	
Jordanians	96 (100.0)
Medical indications, n (%)*	
Preterm birth (< 37-week of gestation)	79 (82.3)
Sepsis (including suspected)	47 (49.0)
IUGR	5 (5.2)
RDS	51 (53.1)
IFC	3 (3.1)
Others (anemia, poor feeding, hypotonia, PUV)	5 (5.2)
Fasting blood Sugar (mg/dl), mean ± SD	83.2 ± 18.8
Triglycerides (mg/dl), mean ± SD	84.8 ± 15.5
SCr (mg/dl), mean ± SD	0.37 ± 0.18
Albumin (mg/dl), mean ± SD	4.05 ± 1.97
[Na +] (mmol/L), mean ± SD	137.7 ± 4.06
[K +] (mmol/L), mean ± SD	5.47 ± 0.91
Blood Urea (mg/dl), median (IQR)	11.3 (7.67–19.1)
CRP (mg/L), median (IQR)	0.90 (0.50–10.5)

*BMI* Body mass index, *CRP* C-reactive protein. IUGR: Intrauterine growth retardation. *IFC* Intrapartum fetal compromise, *PUV* Posterior urethral valves RDS: respiratory distress syndrome, *SCr* Serum creatinine.

*This variable has a total of more than 100% because they are not mutually exclusive.

### TRPs and pharmacists interventions

**Fig. 1:**
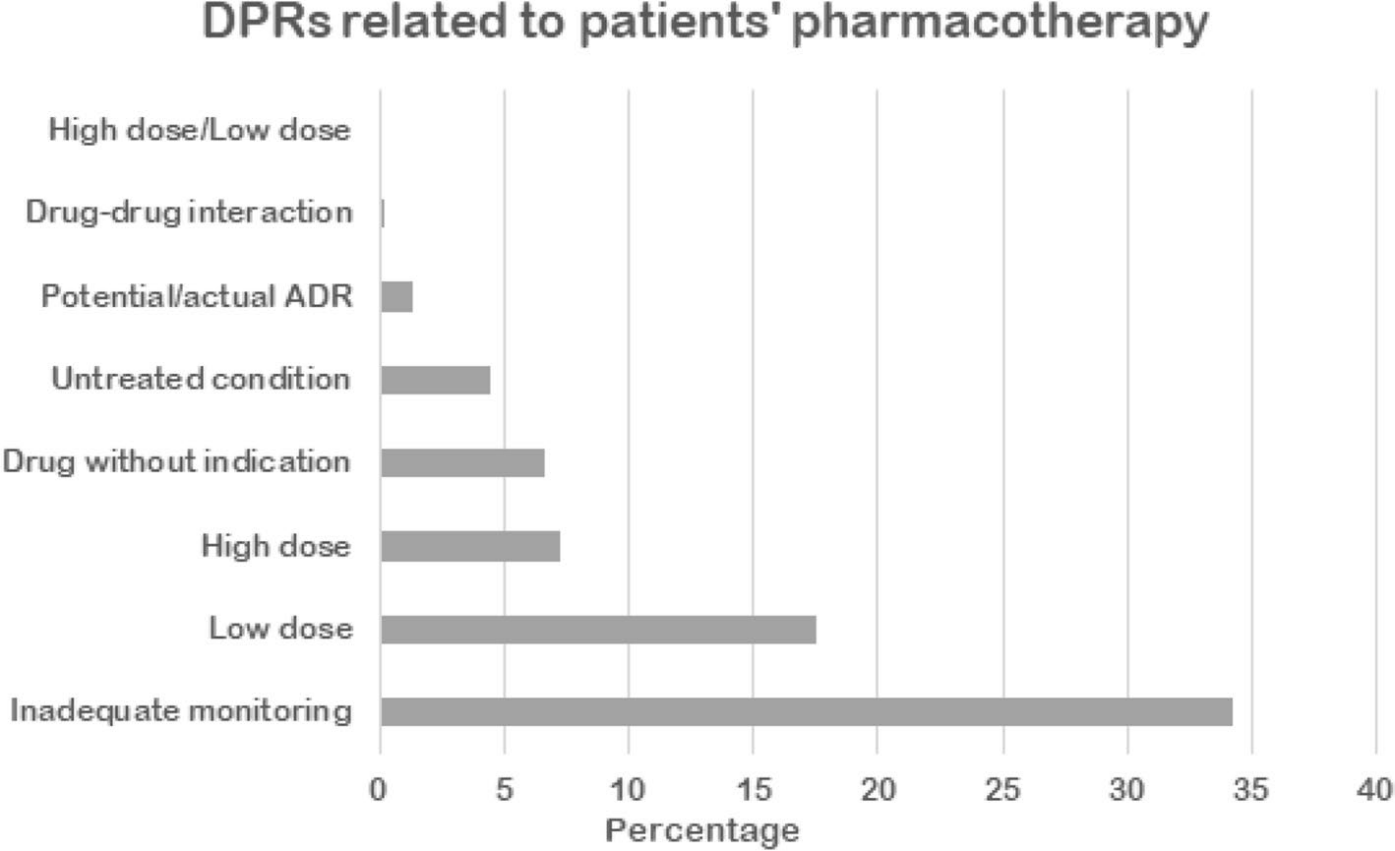
TRPs related to patients’ pharmacotherapy. *ADR* Adverse drug reaction

Neonates received a median (IQR) of 3 (2–5) medications. The median number of disease conditions which required therapy was 2 (1–3) per patient. Patients had a median of 7 TRPs (IQR = 3–13). Among the study sample, a total of 929 TRPs were successfully identified and intervened through by clinical pharmacists, which were subdivided into 3 major groups namely: TRPs related to IV medications preparation, TRPs related to patients’ pharmacotherapy and those related to PN preparation. Detailed information about each subtype of TRPs is illustrated in Table 2 and Fig. 1. We had 27 total TRPs which are related to IV medications (3.0%), the most common of which is drug-IV preparation interaction (*n* = 15, 55.6%), followed by inappropriate infusion rate (*n* = 5, 18.5%). Pharmacists received a total of 57 queries from the nurses regarding IV medication preparations, mostly being regarding IV medication dose/ preparation/administration/ infusion rate (*n* = 41, 71.9%).

**Table 2:** TRPs related to IV and PN preparations as frequency and percentage

**TRPs related to IV preparations with pharmacist intervention**	N	%
Dose is inappropriate	2	7.4
Drug-IV interaction	15	55.6
Infusion rate is inappropriate	5	18.5
Inappropriate diluent	1	3.7
Inappropriate storage	1	3.7
Infusion time not specified	1	3.7
A photosensitive drug was not protected from light after reconstitution	1	3.7
IV medication working concentration written wrongly despite right preparation	1	3.7
Total	**27**	
**IV-related queries with pharmacists’ intervention**		
Asking about IV medication dose/ preparation/administration/ infusion rate	41	71.9
Asking about IV medication photosensitivity	1	1.8
Asking about IV medication stability	1	1.8
Asking about IV medication storage	1	1.8
Asking about medication compatibility	7	12.3
Asking about medication stability	4	7.0
Asking about oral preparation from IV preparation	2	3.5
**Total**	**57**	
**TRPs related to PN preparations with pharmacist’s intervention**		
Drug-PN interaction	3	4.0
Inadequate monitoring	14	18.4
PN composition is not matched with patient’s nutritional needs (nutritional imbalances)	33	43.4
PN was complicated	12	15.8
Total	**62**	
**PN-related queries with pharmacists’ intervention**		
Asking about medication-PN compatibility	3	10.0
Asking about PN preparation/composition	27	20.0
**Total**	**30**

Pharmacotherapy-related TRPs were the most common type (*n* = 839, 90.3%). Pharmacist interventions were documented for all these TRPs. The most common TRPs related to patient’s pharmacotherapy were inadequate monitoring (34.2%, *n* = 287) followed by low dose (17.5%, *n* = 147) (Fig. 1). Inadequate monitoring means insufficient repeated testing for drug levels or relevant laboratory parameters, that are necessary for the safe and effective use of medications. Amongst this category of TRPs, more than 75% (*n* = 233, 77.7%) were related to infrequent therapeutic drug monitoring (TDM) for amikacin and vancomycin, while the rest were related to lack of renal and/or liver function tests assessment, as well as electrolytes and vitamin D levels, when indicated.

As for PN-related TRPs (*n* = 62, 6.7%), the PN composition was not matched with patient’s nutritional needs in more than third of patients (43.2%), which is due to untoward adjustment of the standard formula according to individualized laboratory values by the clinical pharmacist. Inadequate monitoring was documented in (*n* = 14, 15.8%). In 11 of the cases, there was no reassessment of triglycerides levels after the administration of a 20% Intravenous fat emulsion (Intralipid® 20%). In the other 3 cases, there was infrequent monitoring of sodium and potassium in spite of abnormalities in these electrolytes. PN-related complications represented 18.4% of TRPs including metabolic complications (namely, hypertriglyceridemia) and electrolytes abnormalities. In 20% of patients, requests about formulation of the PN preparation were directed and answered by the pharmacist (*n* = 27) (Table 2).

Bivariate correlation testing was then conducted to identify significant associations between the number of TRPs and other single variables. A positive statistical correlation exists between the number of medications, length of hospitalization, with the number of reported TRPs (*r* = 0.275, *p* < 0.01), (*r* = 0.532, *p* < 0.001), respectively. Moreover, the total number of TRPs was statistically and negatively associated with neonatal weight (*r* = -0.282, *p* = 0.005) and gestational age (*r* = -0.287, *p* = 0.006), but positively with blood urea nitrogen (BUN) (*r* = 0.488, *p* = 0.002), and aminotransferase (ALT) (*r* = 0.380, *p* = 0.013). Using multiple linear regression, it was found that the number of medications, duration of hospital stay, BUN and ALT were significant positive predictors of number of TRPs according to results in Table 3.

**Table 3:** Multiple linear regression of factors correlated with number of TRPs

Variables in the model	β	*p*-value	95% Confidence Interval for β	
			Lower Bound	Upper Bound
(Constant)		0.520	-27.64	52.65
Weight	-0.26	0.097	-11.42	1.05
Number of medications	0.31	0.045	0.05	3.91
Blood urea nitrogen	0.63	0.032	0.04	0.85
Duration of hospital stay	0.38	0.018	0.04	0.38
Gestational age	-0.11	0.480	-1.62	0.72
Serum Creatinine	-0.30	0.157	-43.55	7.61
ALT	0.45	0.009	0.30	1.81
AST	-0.232	0.456	-0.493	0.231

The overall acceptance and implementation rate for the stated interventions by physicians was 98.7% (*n* = 1003). Similarly, a low percentage of interventions rejected, all of which were related to patients’ pharmacotherapy, with accepted interventions of 98.6% (*n* = 827) (Table 4).

**Table 4:** Implementation of pharmacists’ interventions related to pharmacotherapy

TRP category	Applied by physician		Total
	No	Yes	
Drug without indication	3	52	55
Drug-drug interaction	0	2	2
High dose	1	60	61
High dose/Low dose	0	1	1
Inadequate monitoring	1	286	287
Low dose	2	145	147
Potential/actual ADR	0	12	12
Untreated condition	1	37	38
Others	3	233	236
Total	11	827	839

## Discussion

Neonates is a group of vulnerable population that are susceptible to many TRPS because they receive off-label drugs and doses that are extrapolated from those of adults [18]. Additionally, they are usually subjected to using multiple medications (≥ 5) for various conditions (polypharmacy) during their hospital stay which leads to a higher incidence of TRPs in NICUs compared to other medical wards [19]. It is noteworthy to mention that the NICU in the University of Jordan follows clinical practice guideline (Neofax® and Lexicomp®) that regulate drugs prescribed to infants. In addition, members of the team were trained on appropriate aseptic techniques for the preparation IV drugs, including PN. The presence of guidelines is essential since lack of reliable sources for credible information that physicians can use leads to prescribing errors [20].

The median hospital stay for neonates in this study was 33 days (21.3–45.8) which was relatively long and correlated to number of TRPs. In fact, longer hospital stay increases the number of medication errors, which explains why premature neonates are more prone to TRPs compared to term neonates [21]. Similarly, in our study there was an association between the number of medications and TRPs; several studies have shown that the incidence of TRPs increased as the number of medications was higher [22, 23]. Leopoldino et al. conducted a longitudinal study in Brazil to assess possible predictors of TRPs in NICU, gestational age, certain medications (alprostadil, antibiotics, and omeprazole) and neurological, cardiac, and renal diseases were risk factors for TRPs in NICUs [24], but neonates on PN were excluded from the latter study. In a recent prospective study of 78 patients, Diaz et al., identified an association between prematurity, number of medications, parenteral nutrition time and negative results associated with medications [25]. Our study showed that most neonates are presented with prematurity so we cannot assess this association. On the other side, the number of TRPS was proportionally correlated with renal and liver damage biomarkers, comparable to what was reported by Leopoldino et al*.* [24].

Neonates have immature organs that influence the pharmacokinetics and pharmacodynamics of drugs. As metabolic pathways mature and infants gain weight, the concentration of drugs continuously change and requires meticulous and regular adjustments [26]. Consequently, adequate dosing for each infant is challenging and highly susceptible to increased toxicities or reduced efficacy [27]. Moreover, an important TRP that is related to IV preparations, which constitute a large majority of drugs administered to neonates, is the stability of these dosage forms [28]. In our study, neonates received a median of 3 medications which is lower than other studies. Leopoldino et al*.* study revealed a higher number of medications received by their cohort, 8.28 ± 6.11 medicines per patient [29].

The classification system used for TRPs was developed and validated in Jordan, while similar studies used different classification systems [29, 30]. The differences in the classification systems might explain the various frequencies of TRPs in studies. A large percentage of TRPs related to patients’ pharmacotherapy was concerned with inadequate monitoring (34.2%). Therapeutic drug monitoring is crucial in neonates due to various developmental changes that produce interindividual variability that necessitates personalized management of drugs [31]. The shift from generalized treatment to individualized approach is important to optimize therapeutic management of neonates [32]. Another TRP that was frequent in our study was dose related, especially low dose (17.5%). This was also revealed in other studies where sub-optimal drug efficacy was predominant [30, 33]. Nunes et al*.* showed that most of the identified TRPs were related to dosing, which indicates a problem in prescription by the physician [34]. Overdosing is also common in neonates, Krzyzaniak et al*.* stated in their review that assessed medication errors in hospitalized patients (including neonates), that 42% of administration errors were either under-dose or overdose [35].

In general, the overall acceptance and implementation rate for the interventions was high (98.72%), which demonstrates the acceptance of clinical pharmacist intervention and the perception that this group of patients requires the intervention of all the members of health care team. It also demonstrates the valuable role that pharmacists can play in preventing TRPs in neonates. Similar studies showed a high acceptance rate of interventions provided by the pharmacists, 93.1% [29].

While many studies evaluated TRPs and associated risk factors in adults receiving PN, one study focused on neonates as vulnerable population [23]. Strengths of the study include using sufficient cohort of neonates from various hospital wards (not only ICU), and the adoption of a standard TRPs classification system. Still, this classification might not be comprehensive in detecting all potential and actual TRPs. We also assessed the extent of acceptance of pharmacy interventions by physicians. However, the retrospective nature of the study, short duration, and the fact that data was collected from one institution, might hinder the study’s generalizability. Longitudinal follow-up studies are necessary to see the effect of pharmacists’ intervention on the PN outcomes in terms of both effectiveness and safety.

## Conclusion

Despite the presence of clinical guidelines, neonates who received PN were exposed to different types of TRPs. Results from this observational study showed that the most common type was pharmacotherapy-related, primarily due to insufficient monitoring frequency as well dosing errors. Most of the recommendations to overcome these TPRs were accepted and implemented by physicians. Thus, Incorporating a clinical pharmacist fully and efficiently into the healthcare team could potentially serve as a proactive approach to decrease the occurrence of these problems.

## Data Availability

Data are available upon request from the corresponding author.
